# 

**Published:** 1828-07

**Authors:** 


### TO SUBSCRIBERS.

THE undersigned, partly in consequence of a declining state of health, has been under the necessity of disposing of the "*Western Journal of the Medical and Physical Sciences.*" The Editor, Dr. Daniel Drake, is the purchaser; and will, hereafter, be the sole Proprietor and Publisher as well as Editor. To him or his authorized agents, therefore, in future, all letters of business must be directed, and all payments remitted.

E. S. BUXTON.

Cincinnati, Ohio, July 31st, 1828.

